# Synthesis of 5-oxyquinoline derivatives for reversal of multidrug resistance

**DOI:** 10.3762/bjoc.8.193

**Published:** 2012-10-05

**Authors:** Torsten Dittrich, Nils Hanekop, Nacera Infed, Lutz Schmitt, Manfred Braun

**Affiliations:** 1 Institute of Organic and Macromolecular Chemistry, University of Düsseldorf, Universitätsstr. 1, D-40225 Düsseldorf, Germany; 2 Institute of Biochemistry, University of Düsseldorf, Universitätsstr. 1, D-40225 Düsseldorf, Germany

**Keywords:** acetals, enantiopure compounds, heterocycles, inhibitor, multidrug resistance

## Abstract

The inhibition of ABC (ATP binding cassette) transporters is considered a powerful tool to reverse multidrug resistance. Zosuquidar featuring a difluorocyclopropyl-annulated dibenzosuberyl moiety has been found to be an inhibitor of the P-glycoprotein, one of the best-studied multidrug efflux pumps. Twelve 5-oxyisoquinoline derivatives, which are analogues of zosuquidar wherein the dibenzosuberyl-piperazine moiety is replaced by either a diarylaminopiperidine or a piperidone-derived acetal or thioacetal group, have been synthesized as pure enantiomers. Their inhibitory power has been evaluated for the bacterial multidrug-resistance ABC transporter LmrCD and fungal Pdr5. Four of the newly synthesized compounds reduced the transport activity to a higher degree than zosuquidar, being up to fourfold more efficient than the lead compound in the case of LmrCD and about two times better for Pdr5.

## Introduction

The treatment of cancer is often severely hampered by efflux pumps, which are responsible for the extrusion of various chemotherapeutics from the tumor cell, an effect termed “multidrug resistance”. Various transporters of the ATP-binding cassette family, so called ABC transporters, have been shown to be responsible for multidrug resistance [1]. Among these multidrug-resistance ABC transporters, the P-glycoprotein has been investigated most intensively [2]. As P-glycoprotein is considered to be a major player in multidrug resistance and has been found to be over expressed in tumor cells [3–4], considerable attempts have been made to develop inhibitors of P-glycoprotein in order to reverse multidrug resistance. These efforts were pursued for a longer period leading to a variety of small-molecular compounds with different structures that act as P-glycoprotein inhibitors [5]. The drug zosuquidar (**1a**) emerged as the most promising among them [6–9]; however, so far it has not made its way into routine clinical application [10].

Apart from their role in multidrug resistance of tumor cells, ABC-type efflux pumps are also involved in multidrug resistance of pathogenic Gram-positive bacteria and fungi for which inhibitors have only rarely been studied. Therefore, we were interested in developing inhibitors of efflux pumps from bacterial and fungal organisms, namely LmrCD from *Lactococcus lactis* and Pdr5 from *Saccharomyces cerevisiae* [11–12]. Thus, we synthesized, guided by the lead structure zosuquidar (**1a**), a series of compounds that were evaluated as potential inhibitors of LmrCD and Pdr5. Whereas biochemical studies with the novel compounds will be disclosed separately [13], we present in this article the synthesis, in detail, of twelve new compounds that may provide reversal of multidrug-resistant activity.

## Results and Discussion

### Synthesis

**Scheme 1:**
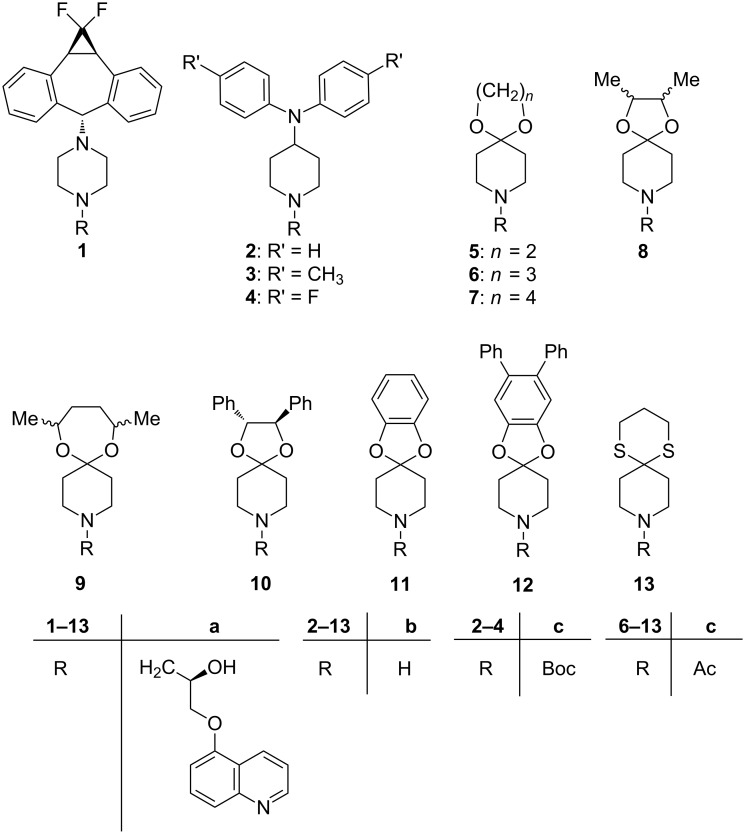
Lead structure of zosuquidar (**1a**) and new inhibitors **2**–**13** (series **a**); precursors **2**–**13** (series **b**); precursors **6**–**13** (series **c**).

As extended studies on modifications of zosuquidar (**1a**) had revealed both the 5-oxyquinoline and the hydroxypropane spacer as being crucial for inhibitory activity on P-glycoprotein [14–18], our strategy was based on modification or replacement of the dibenzosuberyl and the piperazine moieties, whereas the above-mentioned “southern part” of zosuquidar (**1a**) was planned to be maintained, including the absolute configuration of the stereogenic carbinol center. Thus, we replaced the piperazine motif in **1** by a piperidine ring carrying either nitrogen or oxygen as a hetero substituent(s) in the 4-position. Therefore, an initial series of 4-aminopiperidines **2a**–**4a** and, in a second approach, new spirocyclic acetals **5a**–**12a** as well as the thioacetal **13a** were synthesized. All the new compounds, shown in Scheme 1, were obtained in enantiomerically pure form, with the stereogenic carbinol center adopting (*R*) configuration. The acetals **8a** and **9a** formed as mixtures of diastereomers, whereas compound **10a** was enantiomerically and diastereomerically pure.

**Scheme 2:**
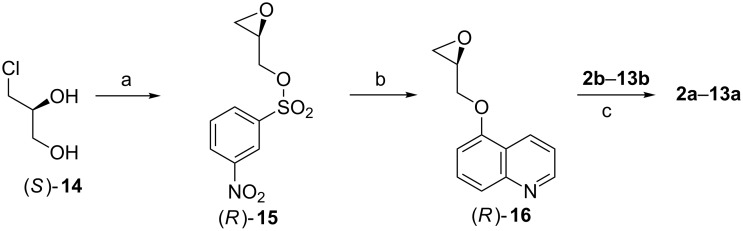
Synthetic route to compounds **2a**–**13a**. Reagents and conditions: (a) K_3_PO_4_, CH_2_Cl_2_, reflux, 3 h; then triethylamine, 0 °C, 1 h; then: 3-nitrobenzenesulfonyl chloride, 0 to 25 °C, 1 h; 72%. (b) 5-hydroxyquinoline, K_2_CO_3_, DMF, 25 °C, 20 h; 67%. (c) EtOH, reflux, 3 h; **2a**: 72%; **3a**: 72%; **4a**: 69%; **5a**: 83%; **6a**: 79%; **7a**: 77%; **8a**: 98%; **9a**: 61%; **10a**: 83%; **11a**: 72%; **12a**: 90%; **13a**: 29%.

The commercially available chiral building block (*S*)-3-chloro-1,2-propanediol (**14**) was chosen as the starting material. It was converted to (*R*)-nosylate **15** in three steps by following a modified procedure [19], as shown in Scheme 2. The subsequent treatment with 5-hydroxyquinoline [20] led to the epoxide (*R*)-**16**, serving as the chiral building bloc in the final key step, which involves the nucleophilic attack of the secondary amino group of the precursors **2b**–**13b** to the epoxide **16**, to deliver the final products **2a**–**13a**. The yields of the final coupling step are given in Scheme 2.

**Scheme 3:**
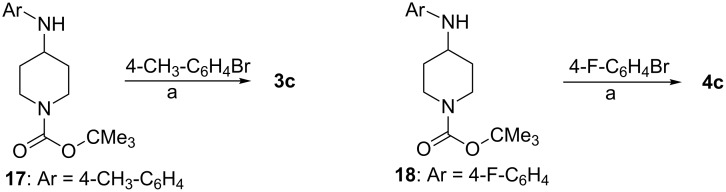
Preparation of *N*-Boc-protected 4-aminopiperidines **3c** and **4c**. Reagents and conditions: (a) NaO*t-*Bu, Pd(OAc)_2_, *t*-Bu_3_P, toluene, reflux, 16 h; **3c**: 76%; **4c**: 41%.

The precursor **2c** was obtained by an N-arylation, as described in the literature.[21] A modified Buchwald–Hartwig amination reaction using tri-*tert*-butylphosphane as a ligand at palladium [22] was applied in order to couple 4-methylphenyl bromide and 4-fluorophenyl bromide with Boc-protected aminopiperidines **17** and **18**, respectively. Thus, diarylaminopiperidine **3c** and **4c** became readily accessible, as outlined in Scheme 3. The cleavage of the protecting group occurred according to standard protocols by treatment with trifluoroacetic acid to liberate the amines **2b**–**4b**, which were used without further purification in the coupling reaction with epoxide (*R*)-**16**.

The spirocyclic compounds **6c**–**13c** were obtained by standard acid-catalyzed reaction with the corresponding diols for the formation of acetals **6c**–**12c** and propanedithiol for **13c**, starting from *N*-acetyl-4-piperidone. Basic hydrolysis was found to be appropriate for the removal of the N-protecting group. Again, the free amines **6b**–**13b** were used as crude products for the reaction with the oxirane (*R*)-**16** to give the series of new compounds. Spiroacetal **5b** was commercially available.

### Biochemical studies

The transport activity [13] of the compounds **2a**–**13a** and the reference compound zosuquidar (**1a**) was determined for the ABC transporters LmrCD and Pdr5. The results are given in Table 1, which restricts itself to those compounds that displayed a significantly enhanced activity compared to that of **1a**. The activity of the individual transporters in the absence of either zosuquidar (**1a**) or any of the new compounds **2a**–**13a** is assigned as 100%, meaning that those substances that show a lower relative transport activity than zosuquidar (**1a**) are considered as potential improvements. Various new 5-oxyquinoline derivatives show activity that is comparable or inferior to that of **1a**. For reasons of clarity, only those results are included in Table 1 that indicate a substantially higher reduction of transport activity than that for zosuquidar (**1a**). It turns out that, at the transporter LmrCD (first column), a derivative with a 4-aminopiperidine skeleton, compound **3a**, was distinctly more active than zosuquidar (**1a**). Moreover, the acetal derivative **10a** was found to be about four times more active than the lead compound **1**. For the fungal transporter Pdr5 (second column), one compound of the 4-aminopiperidine series, **2a**, is about twice as active as zosuquidar (**1a**). Similar activity is provided by catechol-derived acetal **13a**, whereas, at this transporter, the acetals derived from 1,2-diphenylethanediol, are still active as transport inhibitors, but their activity is slightly lower than that of zosuquidar (31% relative transport activity). The full details of the results of transport inhibition for all the compounds **1a** and **2a** through **13a** are given in a separate communication [13].

**Table 1:** Relative transport activity of LmrCD and Pdr5 in the presence of zosuquidar (**1a**) and selected new 5-oxyquinoline derivatives (**2a**–**13a**).^a^

Compound / Relative transport activity at LmrCD	Compound / Relative transport activity at Pdr5

**1a /** 42.4% **3a /** 19.8% **10a /** 11.2%	**1a /** 23.7% **2a /** 14.0% **11a /** 12.5%

^a^100% activity corresponds to the transport activity in the absence of either zosuquidar (**1a**) or any 5-oxyquinoline derivative. The reported values represent the average of three independent measurements.

## Conclusion

The replacement of the benzosuberyl moiety of zosuquidar (**1a**) by diarylamino or acetal groups lead to a series of twelve new compounds, which have been tested as potential inhibitors of the multidrug-resistance ABC transporters LmrCD and Pdr5. As a result, four of the new compounds were identified as being superior to zosuquidar in terms of transport reduction. The compound **10a**, which was found to be the most efficient of all, features a cyclic acetal moiety derived from (*R*,*R*)-1,2-diphenyl-1,2-ethanediol.

## Experimental

**General:** Melting points were determined with a Büchi 540 melting point apparatus and are not corrected. NMR spectra were recorded in CDCl_3_ with a Bruker Avance DRX 200 and a Bruker Avance DRX 500 spectrometer. IR spectra were measured with a Bruker Vector 22 spectrometer. Mass spectra were recorded on a Thermo Finnigan Trace DSQ apparatus (GCMS), an ion-trap API mass spectrometer Finnigan LCQ Deca (ESI), a triple-quadrupole-mass spectrometer Finnigan TSQ 7000 (EI), and a sector field mass spectrometer Finnigan MAT 8200 (EI, 70 eV). High-resolution mass spectroscopy was carried out on Bruker FT-ICR APEX III (7.0 T) (MALDI) at the University of Bielefeld. Column chromatography was performed with Fluka silica gel 60 (230–400 mesh), and thin-layer chromatography was carried out by using Merck TLC silicagel 60 F_254_ aluminium sheets. Toluene was freshly distilled from sodium under nitrogen.

### General procedure for the reaction of piperidines **2b**–**13b** with (*R*)-**16**

A solution of (*R*)-**16** (0.101 g, 0.50 mmol) and a piperidine **2b**–**13b** (0.50 mmol) in ethanol (10 mL) was heated under reflux in a 25 mL flask for 3 h. After cooling to room temperature, the solvent was removed in a rotary evaporator, and the residue was purified by column chromatography on silica gel (chloroform/methanol, 10:1). Thus, the products **2a**–**13a** were obtained as white or yellowish solids or oils.

### General procedure for the preparation of acetals **6c**–**12c**

Under nitrogen, a mixture of *N*-acetyl-4-piperidinone (1.13 g, 8.0 mmol), the corresponding diol (or dithiol), and *p*-toluenesulfonic acid monohydrate (0.095 g, 0.5 mmol) in chloroform (60 mL) was heated under reflux in a 100 mL flask, equipped with a Soxhlet extraction apparatus, which was filled with molecular sieves (4 Å). After heating under reflux for 10 to 15 h, the mixture was cooled to room temperature, a saturated aqueous solution of sodium hydrogen carbonate (20 mL) was added, and the mixture was stirred for 10 min at room temperature. The aqueous layer was removed, and the organic layer was dried with sodium sulfate. After removal of the solvent in a rotary evaporator, the residue was purified by flash chromatography. For the preparation of catechol-derived acetals **11c** and **12c**, toluene was used instead of chloroform, and trifluoromethanesulfonic acid instead of *p*-toluenesulfonic acid.

### General procedure for the N-arylation of *N*-Boc-4-arylaminopiperidines **17** and **18**

A 25 mL two-necked flask, equipped with a magnetic stirrer and a connection to a combined argon/vacuum line was charged with piperidine **19** or **20** (3.6 mmol), the corresponding aryl bromide (3.6 mmol), sodium *tert*-butoxide (0.43 g, 4.5 mmol) and palladium acetate (10.1 mg, 0.045 mmol). The flask was closed with a septum and the air in the flask was replaced by nitrogen. Dry toluene (5 mL) was added, and the mixture was stirred at room temperature for 30 min. Then, a solution of tri-*tert*-butylphosphane (0.12 mL, 0.5 mmol) in toluene (1 mL) was added, and the mixture was refluxed for 16 h under argon. After cooling to room temperature, water (10 mL) was added, and the organic layer was separated, dried with sodium sulfate and evaporated. The crude product was purified by column chromatography (ethyl acetate/cyclohexane, 1:2) to give the yellowish solid products.

## Supporting Information

File 1 Procedures. Spectroscopic and analytical data.
