# Supplementary material for: Synthesis of 5-oxyquinoline derivatives for reversal of multidrug resistance
Source: Beilstein J Org Chem. 2012 Oct 5;8:1700–4. doi: 10.3762/bjoc.8.193 (PMC3511002; doi:10.3762/bjoc.8.193)
Supplement: File 1 — Procedures. Spectroscopic and analytical data. [file Beilstein_J_Org_Chem-08-1700-s001.pdf]

Supporting Information

for

Synthesis of 5-oxyquinoline derivatives for reversal of multidrug resistance

Torsten Dittrich[1], Nils Hanekop[2], Nacera Infed[2], Lutz Schmitt[2] and Manfred Braun*[1]

Address: [1]Institute of Organic and Macromolecular Chemistry and [2]Institute of Biochemistry, University of Düsseldorf, Universitätsstr. 1, D-40225 Düsseldorf, Germany

Email: Manfred Braun* - braunm@hhu.de

* Corresponding author

Procedures. Spectroscopic and analytical data

General procedure for the reaction of piperidines 2b–13b with (*R*)-16:

A solution of (*R*)-**16** (0.101 g, 0.50 mmol) and a piperidine **2b**–**13b** (0.50 mmol) in ethanol (10 mL) was heated under reflux in a 25 mL flask for 3 h. After cooling to room temperature, the solvent was removed in a rotary evaporator, and the residue was purified by column chromatography on silica gel (chloroform/methanol, 10:1). Thus, products **2a**–**13a** were obtained as white or yellowish solids or oils. According to this procedure, the following were obtained:

(2*R*)-1-[4-(Diphenylamino)piperidin-1-yl]-3-(quinolin-5-yloxy)propan-2-ol (**2a**):

Prepared from (*R*)-**16** and piperidine **2b**. Yield: 72%; $[\alpha]_D^{20}$ –6.0 (*c* =1, ethanol); R_f = 0.8; ^{1}H NMR (500 MHz): δ = 1.42–1.60 (m, 2H), 1.98 (d, *J* = 10.7 Hz, 2H), 2.18 (t, *J* = 11.7 Hz, 1H), 2.46 (t, *J* = 12.0 Hz, 1H), 2.54–2.63 (m, 2H), 2.94 (d, *J* = 11.7 Hz, 1H), 3.10 (d, *J* = 11.4 Hz, 1H), 3.85–3.93 (m, 1H), 4.06–4.20 (m, 3H), 6.81–6.78 (m, 5H), 7.00 (t, *J* = 7.6 Hz, 2H), 7.25–7.30 (m, 4H), 7.30–7.34 (m, 1H), 7.55 (t, *J* = 8.5 Hz, 1H), 7.69 (1H, d, *J* = 8.5 Hz, 1H), 8.45 (dd, *J* = 8.2 Hz, *J* = 1.6 Hz, 1H), 8.85–8.88 (m, 1H); ^{13}C NMR (125 MHz): δ = 32.80, 52.06, 58.5, 61.19, 66.10, 71.31, 105.74, 113.72, 117.81, 120.67, 121.26, 122.21, 129.78, 129.83, 131.29, 147.37, 149.39,151.06, 154.55; ESIMS: *m*/*z* = 454 (100) $[M + H]^+$, 285 (15).

(2*R*)-1-{4-[Bis(4-methylphenyl)amino]piperidin-1-yl}-3-(quinolin-5-yloxy)propan-2-ol (**3a**):

Prepared from (*R*)-**16** and piperidine **3b**. Yield: 72%; mp 144.2–144.5 °C; $[\alpha]^D_{20}$ –9.1 (*c* = 1, ethanol); R_f = 0.81; IR (KBr): $\tilde{\nu}$ = 3261, 2937, 2786, 1598, 1507, 1276, 1243, 1099, 794 cm^{-1}; ^{1}H NMR (500 MHz): δ = 1.34–1.52 (m, 2H), 1.88 (d, *J* = 11.0 Hz, 2H), 2.10 (t,

J = 10.7 Hz, 1H), 2.21 (s, 6H), 2.38 (t, *J* = 11.7 Hz, 1H), 2.46–2.56 (m, 2H), 2.85 (d, *J* = 10.7 Hz, 1H), 3.02 (d, *J* = 11.4 Hz, 1H), 3.71–3.81 (m, 1H,), 3.96–4.12 (m, 3H,) 6.64 (d, *J* = 8.0 Hz, 4H), 6.73 (d, *J* = 7.9 Hz, 1H), 6.98 (d, *J* = 8.20, 4H), 7.21–7.26 (m, 1H), 7.46 (t, *J* = 7.9 Hz, 1H), 7.60 (d, *J* = 8.8 Hz, 1H), 8.45 (d, *J* = 8.5 Hz, 1H), 8.77 (1H, d, *J* = 4.1 Hz, 1H); ^{13}C NMR (125 MHz): δ = 19.61, 28.95, 53.49, 54.14, 59.75 , 64.54 , 69.77, 104.31, 119.18, 119.78, 120.54, 121.66, 128.45,128.81, 130.25, 142.74, 147.72, 149.44, 153.01, 175.69; ESIMS *m*/*z* (%): 482 (100) $[M + H]^+$; HRMS $[C_{31}H_{36}N_3O_2]$: calcd. 482.2795; found 482.2802.

(2*R*)-1-{4-[Bis(4-Fluorophenyl)amino]piperidin-1-yl}-3-(quinolin-5-yloxy)propan-2-ol (**4a**):

Prepared from (*R*)-**16** and piperidine **4b**. Yield: 69%; $[\alpha]^{D}_{20}$ – 8.6° (*c* = 1, ethanol); R_f = 0.49; IR (KBr): $\tilde{\nu}$ = 3422, 2972, 1618, 1589, 1501, 1467, 1265, 1212, 1093, 769, 476 cm^{-1}; ^{1}H NMR (500 MHz): δ = 1.32–1.48 (m, 2H), 1.87 (d, *J* = 11.0 Hz, 2H), 2.10 (t, *J* = 10.7 Hz, 1H), 2.38 (t, *J* = 11.7 Hz, 1H), 2.48–2.57 (m, 2H), 2.82–2.90 (m, 1H), 3.02 [d, *J* = 10.1 Hz, 1H), 3.70 (dt, J_t = 11.7 Hz, J_d = 3.47 Hz 1H), 3.98–4.13 (m, 3H), 6.68 (d, *J* = 9.1 Hz, *J* = 4.7 Hz), 6.75 (dd, *J* = 7.6 Hz, *J* = 3.2 Hz, 1H), 6.89 (t, *J* = 8.5 Hz, 4H), 7.23–7.31 (m, 1H), 7.42–7.51 (m, 1H), 7.61 (t, *J* = 8.5, 1H), 8.43 (d, *J* = 8.5 Hz), 8.8 (dd, *J* = 4.4 Hz, *J* = 1.6 Hz, 1H); ^{13}C NMR (125 MHz): δ = 29.49, 29.80, 54.17. 54.40, 59.63, 64.77, 68.26 , 69.83, 104.26, 114.83, 119.17, 120.83, 123.02, 124.19, 128.32, 129.73, 141.41, 147.99, 149.64, 153.45, 156.45, 158.38; ESIMS: *m*/*z* (%) = 490 (100) $[M + H]^+$, 345 (24), 285 (17); HRMS $(C_{29}H_{30}N_3O_2F_2)$: calcd. 490.2301; found 490.2301.

(2*R*)-1-(1,4-Dioxa-8-azaspiro[4.5]decan-8-yl)-3-(quinolin-5-yloxy)propan-2-ol (**5a**): Prepared from (*R*)-**16** and acetal **5b**. Yield: 83%; $[\alpha]^{D}_{20}$ –7.5 (*c* = 1, ethanol); R_f = 0.25; IR (neat): $\tilde{\nu}$ = 3415, 2952, 2819, 1589, 1470, 1267, 1145, 1094, 798 cm^{-1}; ^1H NMR (500 MHz): δ = 1.73–1.84 (m, 4H), 2.55–2.62 (m, 2H), 2.64–2.69 (m, 2H), 2.79–2.86 (m, 2H), 3.97 (s, 4H), 4.13–4.27 (m, 3H), 6.88 (d, *J* = 7.3 Hz, 1H), 7.35–7.39 (m, 1H), 7.60 (t, *J* = 8.2 Hz, 1H), 7.70 (d, *J* = 8.5 Hz, 1H), 8.56–8.59 (m, 1H), 8.85 (dd, *J* = 4.1 Hz, *J* = 1.58 Hz, 1H); ^{13}C NMR (125 MHz): δ = 35.35, 52.04, 60.62, 64.62 , 66.09, 71.27, 105.67, 107.32, 120.64, 121.26, 122.31, 129.73, 131.19, 149.48, 151.11, 154.57; ESIMS: *m*/*z* = 345 (100) $[M + H]^+$, 200 (28), 188 (9), 156 (12); HRMS ($C_{19}H_{25}N_2O_4$): calcd. 345.1802; found 345.1809.

(2*R*)-1-(1,5-Dioxa-9-azaspiro[5.5]undecan-9-yl)-3-(quinolin-5-yloxy)propan-2-ol (**6a**): Prepared from (*R*)-**16** and acetal **6b**. Yield: 79%; $[\alpha]^{D}_{20}$ –6.8 (*c* = 1, ethanol); R_f: 0.14; IR (neat): $\tilde{\nu}$ = 3375, 2961, 2871, 2823, 1619, 1598, 1470, 1267, 1145, 1099, 1059, 799, 754 cm^{-1}; ^1H NMR (500 MHz): δ = 1.69 (quint, *J* = 5.39 Hz, 2H), 1.94 (s, 4H), 2.48–2.56 (m, 2H), 2.62–2.68 (m, 2H), 2.70–2.77 (m, 2H), 3.87 (t, *J* = 5.7 Hz, 4H), 4.08–4.18 (m, 2H), 4.21–4.27 (m, 1H), 6.83 (1H, d, *J* = 7.6 Hz, 1H), 7.31–7.35 (m, 1H), 7.55 (t, *J* = 8.2 Hz, 1H), 7.66 (d, *J* = 8.5 Hz, 1H), 8.52–8.56 (m, 1H), 8.85 (dd, *J* = 4.10 Hz, *J* = 1.58 Hz,1H); ^{13}C NMR (125 MHz): δ = 26.00, 33.29, 50.59, 58.71, 60.59, 65.98, 71.29, 96.44, 105.66, 120.65, 121.27, 122.31, 129.75, 131.21, 149.49, 151.14, 154.58; ESIMS: *m*/*z* = 359 (100) $[M + H]^+$, 214 (23); HRMS: ($C_{20}H_{27}N_2O_4$): calcd. 359.1965; found 359.1964.

(2*R*)-1-(7,12-Dioxa-3-azaspiro[5.6]dodecan-3-yl)-3-(quinolin-5-yloxy)propan-2-ol (7a):

Prepared from (*R*)-**16** and acetal **7b**. Yield: 77%; $[\alpha]^{D}_{20}$ –5.7 (*c* = 1, ethanol); R_f = 0.34; IR (neat): $\tilde{\nu}$ = 3362, 2941, 1619, 1589, 1469, 1407, 1364, 1315, 1267, 1144, 1099, 1061, 799, 754 cm^{-1}; ^{1}H NMR (500 MHz): δ = 1.58 (s, 4H), 1.47–1.85 (m, 4H), 2.52–2.60 (m, 2H), 2.64–2.71 (m, 2H), 2.73–2.80 (m, 2H), 3.67 (s, 4H), 4.08–4.19 (m, 2H), 4.23–4.29 (m, 1H), 6.83 (d, *J* = 7.9 Hz, 1H), 7.31–7.35 (m, 1H), 7.56 (t, *J* = 8.2 Hz, 1H), 7.67 (d, *J* = 8.5 Hz, 1H), 8.53–8.56 (m, 1H), 8.9 (dd, *J* = 4.10 Hz, *J* = 1.6 Hz, 1H); ^{13}C NMR (125 MHz): δ = 30.04, 34.26, 51.34, 58.47, 62.13, 66.11, 71.33, 99.58, 105.66, 120.59, 121.24, 122.10, 129.78, 131.31, 149.34,150.99, 154.56; ESIMS. *m*/*z* (%) = 373 (100) $[M + 1]^+$, 301 (17), 283 (2) $[C_{17}H_{20}N_2O_2]$, 156 (28); HRMS: ($C_{21}H_{29}N_2O_4$): calcd. 373.2116; found 373.2122.

(2*R*)-1-(2,3-Dimethyl-1,4-dioxa-8-azaspiro[4.5]decan-8-yl)-3-(quinolin-5-yloxy) propan-2-ol (8a):

Prepared from (*R*)-**16** and acetal **8b** (diastereomeric mixture). Yield: 98%; $[\alpha]^{D}_{20}$ –6.8 (*c* = 1, ethanol), R_f = 0.27; IR (neat): $\tilde{\nu}$ = 3370, 2930, 2819, 1619, 1598, 1470, 1377, 1311, 1266, 1204, 1096, 941, 798, 753 cm^{-1}; ^{1}H NMR (500 MHz): δ =1.07 (d, *J* = 8.5, 6H), 1.64–1.79 (m, 4H), 2.43–2.60 (m, 4H), 2.66–2.79 (m, 2H), 4.03–4.19 (m, 5H), 6.78 (d, *J* = 7.9 Hz, 1H), 7.25–7.29 (m, 1H), 7.50 (t, *J* = 8.2 Hz, 1H), 7.61 (d, *J* = 8.5 Hz), 8.47–8.50 (m, 1H), 8.81 (dd, 1H, *J* = 4.4 Hz, *J* = 1.9 Hz); ^{13}C NMR (125 MHz): 15.99, 35.37, 38.29, 60.96, 66.10, 71.31, 74.11, 105.68, 105.99, 120.60, 121.36, 122.18, 129.76, 131.24, 149.40, 151.04, 154.56; ESIMS: *m*/*z* (%) = 373 (100) $[M + 1]^+$, 228 (19),156 (33); HRMS: ($C_{21}H_{29}N_2O_4$): calcd. 373.2116; found 373.2122.

(2*R*)-1-(8,11-Dimethyl-7,12-dioxa-3-azaspiro[5.6]dodecan-3-yl)-3-(quinolin-5-yloxy) propan-2-ol (**9a**):

Prepared from (*R*)-**16** and acetal **9b** (mixture of diastereomers). Yield: 61%; $[\alpha]^{D}_{20}$ –5.3 (*c* = 1, ethanol); R_f: 0.36; IR (neat): $\tilde{\nu}$ = 3362, 2967, 2930, 2812, 1589, 1496, 1375, 1266, 1092, 797, 754 cm^{-1}; ^1H NMR (500 MHz): δ = 1.11–1.24 (m, 10H), 1.50–1.59 (m, 4H), 2.50 (br. s, 2H), 2.57–2.63 (m, 2H), 2.86–2.76 (m, 2H), 3.91–3.99 (m, 2H), 4.04–4.24 (m, 4H), 6.83 (d, *J* = 7.9 Hz, 1H), 7.30–7.34 (m, 1H), 7.53–7.57 (m, 1H), 7.66 (d, *J* = 8.5 Hz,1H), 8.53–8.57 (m, 1H), 8.85 (dd, *J* = 4.4 Hz, *J* = 1.9 Hz, 1H); ^{13}C NMR (125 MHz, $CDCl_3$): δ = 22.30, 32.51, 36.59, 51.63, 58.48, 65.98, 68.09, 71.39, 99.49, 105.66, 120.59, 121.25, 122.13, 129.78, 131.31, 149.37, 151.01, 154.58; ESIMS: *m*/*z* (%) = 401 (100) $[M + H]^+$, 301 (67), 256 (15), 231; HRMS ($C_{23}H_{33}N_2O_4$): calcd. 401.2435; found 401.2433.

(2*R*)-1-{(2*R*,3*R*)-2,3-Diphenyl-1,4-dioxa-8-azaspiro[4.5]decan-8-yl}-3-(quinolin-5-yloxy)propan-2-ol (**10a**):

Prepared from (*R*)-**16** and (*R,R*)-acetal **10b**. Yield: 83%; mp 73.1–73.6°C; $[\alpha]^{D}_{20}$ –30.7° (c = 1, ethanol); R_f = 0.44; IR (KBr): $\tilde{\nu}$ = 3388, 2926, 2805, 1618, 1589, 1578, 1450, 1409, 1304, 1267, 1145, 1097, 1007, 797, 743, 705 cm^{-1}; ^1H NMR (500 MHz): δ = 2.08–2.16 (m, 4H), 2.68–2.80 (m, 4H), 2.95–3.03 (m, 2H), 4.14–4.28 [m, 2H), 4.29 (sext, *J* = 4.7 Hz, 1H), 4.78 (s, 2H), 6.87 (d, *J* = 7.6 Hz, 1H), 7.21–7.24 (m, 4H), 7.29–7.40 (m, 7H), 7.59 (t, *J* = 8.2 Hz, 1H), 7.71 (d, *J* = 8.5 Hz, 1H), 8.57–8.61 (m 1H), 8.88 (dd, *J* = 4.1 Hz, *J* = 1.6 Hz, 1H); ^{13}C NMR (125 MHz, $CDCl_3$): δ = 36.75, 51.92, 60.71, 66.13, 71.24, 85.68, 105.73, 120.68, 121.27, 122.26, 127.12, 127.45, 128.44, 129.81, 131.32,

136.95, 140.68, 149.42, 151.10, 154.55; ESIMS *m*/*z* (%): 497 (100) $[M + H]^+$, 301 (25); HRMS: ($C_{31}H_{33}N_2O_4$): calcd. 497.2340; found 497.2435.

(2*R*)-1-(Quinolin-5-yloxy)-3-[spiro(1,3-benzodioxole-2,4´-piperidin)-1´-yl]propan-2-ol (**11a**):

Prepared from (R)-**16** and acetal **11b**. Yield: 72%; mp 118.2–118.6 °C; $[\alpha]^{D}_{20}$ –4.4 (*c* = 1, ethanol); R_f = 0.38; IR (KBr): $\tilde{\nu}$ = 3382, 2931, 2822, 1619, 1589, 1486, 1406, 1356, 1267, 1239, 1097, 1064, 789, 795 cm^{-1}; ^1H NMR (500 MHz): δ =2.02–2.14 (m, 4H), 3.66–2.76 (m, 4H), 2.88–2.96 (m, 2H), 4.14–4.23 (m, 2H), 4.23–4.30 (m, 1H), 6.75–6.81 (m, 4H), 6.87 (m, 1H), 7.34 (m, 1H), 7.85 (t, *J* = 7.6 Hz, 1H), 7.71 (d, *J* = 8.5 Hz), 8.57 (d, *J* = 8.5 Hz, 1H), 8.87–8.89, (m, 1H); ^{13}C NMR (125 MHz): δ = 35.38, 51.19, 58.64, 66.33, 71.21, 105.72, 109.09, 116.13, 120.24, 121.67, 122.8, 129.81, 131.25, 147.37, 149.39, 151.07, 154.51; ESIMS: *m*/*z* (%) = 393 (67) $[M + H]^+$, 283 (13), 248 (100); HRMS: ($C_{23}H_{25}N_2O_4$): calcd. 393.1801; found 393.1809.

(2*R*)-1-[5,6-Diphenylspiro(1,3-benzodioxole)-2,4’-piperidine)-1’-yl]-3-(quinoline-5-yloxy)propan-2-ol (**12a**):

Prepared from (*R*)-**16** and acetal **12b**. Yield: 90%; $[\alpha]^{D}_{20}$ –8.2 (*c* = 1, ethanol); IR (KBr): $\tilde{\nu}$ = 3423, 2926, 1618, 1589, 1482, 1267, 1237, 1213, 1097, 1065, 789, 771, 701 cm^{-1}; ^1H NMR (500 MHz): δ = 2.11–2.23 (m, 4H), 2.76 (d, *J* = 6.6 Hz, 4H), 2.93–3.01 (m, 2H), 4.17–4.33 (m, 3H), 6.86 (s, 2H), 6.89 (d, *J* = 7.6 Hz, 1H), 7.07 (dd, *J* = 7.6 Hz, *J* = 1.9 Hz, 4H) 7.13–7.21 (m, 6H), 7.36–7.40 (1H, m, 1H), 7.61 (t, *J* = 8.2 Hz, 1H), 7.72 (d, *J* = 8.5 Hz, 1H), 8.58–8.61 (m, 1H), 8.91 (dd, *J* = 4.1 Hz, *J* = 1.9 Hz, 1H); ^{13}C NMR (125 MHz): δ = 35.50, 45.05, 60.57, 66.32, 71.34, 105.68, 111.03, 117.10, 120.69,

121.25, 122.37, 126.61, 128.25, 129.79, 130.42, 131.20, 134.48, 141,93, 146.96, 151.15, 154.52; ESIMS: *m*/*z* (%) = 545 (100) $[M + H]^+$, 454 (23), 400 (52); HRMS: ($C_{35}H_{33}N_2O_4$): calcd. 545.2435; found 545.2434.

(2*R*)-1-(1,5-Dithia-9-azaspiro[5.5]decan-9-yl)-3-(quinolin-5-yloxy)-propan-2-ol (**13a**): Prepared from (*R*)-**16** and thioacetal **13b**. Yield: 29%; $[\alpha]^{D}_{20}$ –10.1 (*c* = 1, ethanol); R_f: 0.53; IR (neat): $\tilde{\nu}$ = 3357, 2940, 2822, 1589, 1468, 1407, 1267, 1096, 798, 752 cm^{-1}; ^1H NMR (500 MHz): δ = 1.95–2.01 (m, 2H), 2.07–2.19 (m, 4H), 2.56–2.69 (m, 4H), 2.76–2.86 (m, 6H), 4.09–4.14 (m,1H), 4.15–4.25 (m, 2H), 6.85 (d, *J* = 7.6 Hz, 1H), 7.31–7.37 (m, 1H), 7.57 (t, *J* = 8.5 Hz, 1H), 7.68 (d, *J* = 8.5 Hz, 1H), 8.53–8.57 (m, 1H), 8.87 (dd, *J* = 4.4 Hz, *J* = 1.9 Hz, 1H); ^{13}C NMR (125 MHz): δ = 26.22, 26.30, 37.99, 48.35, 49.84, 60.97, 65.94, 71.22, 105.68 , 120.63, 121.24, 122.23, 129.81, 131.24, 149.40, 151.07, 154.54; ESIMS: *m*/*z* (%) = 391 (100) $[M + H]^+$, 246 (28); HRMS: ($C_{20}H_{27}N_2O_2S_2$): calcd. 391.1502; found 391.1509.

General procedure for the preparation of acetals 6c–12c:

Under nitrogen, a mixture of *N*-acetyl-4-piperidinone (1.13 g, 8.0 mmol), the corresponding diol (or dithiol), and p-toluenesulfonic acid monohydrate (0.095 g, 0.5 mmol) in chloroform (60 mL) was heated under reflux in a 100 mL flask, equipped with a Soxhlet extraction apparatus, which was filled with molecular sieves (4 Å). After heating under reflux for 10 to 15 h, the mixture was cooled to room temperature, a saturated aqueous solution of sodium hydrogen carbonate (20 mL) was added, and the mixture was stirred for 10 min at room temperature. The aqueous layer was removed, and the organic layer was dried with sodium sulfate. After removal of the solvent in a

rotary evaporator, the residue was purified by flash chromatography. For the preparation of catechol-derived acetals **11c** and **12c**, toluene was used instead of chloroform, and trifluoromethanesulfonic acid instead of *p*-toluenesulfonic acid. According to this procedure, the following compounds were obtained:

1-(1,5-Dioxa-9-azaspiro-[5.5]uncan-9-yl)ethan-1-one (**6c**):

Prepared from 1,3-propandiol. Yield: 81%; mp 71.0–71.6 °C; IR (KBr): $\tilde{\nu}$ = 2955, 2882, 1633, 1447, 1395, 1254, 1146, 1108, 1048, 1004, 969, 889 cm^{-1}; ^1H NMR (500 MHz): δ = 1.62–1.80 (m, 2H), 1.81–1.88 (m, 4H), 2.08 (s, 3H), 3.44 (dd, *J* = 5.7 Hz, *J* = 6.0 Hz, 2H), 3.59 (dd, *J* = 5.7 Hz, *J* = 6.0 Hz, 2H), 3.83–3.95 (m, 4H); ^{13}C NMR (500MHz): δ = 21.85, 32.02, 34.75, 38.54, 43.41, 59.81, 96.45, 169.27; GCMS *m*/*z* (%): 199 (50) [M^+], 156 (30), 140 (20), 113 (100), 100 (20), 98 (15); anal. calcd. for $C_{10}H_{17}NO_3$: C 60.28, H 8.60, N 7.03; found: C 60.17, H 8.86, N 6.70.

1-(7,12-Dioxa-3-aza-spiro[5.6]dodecan-3-yl)ethan-1-one (**7c**):

Prepared from 1,4-butandiol. Yield: 76%; mp 57.7–58.2°C; IR (KBr): $\tilde{\nu}$ = 2940, 1647, 1448, 1357, 1245, 1116, 1058 cm^{-1}; ^1H NMR (500 MHz): δ = 1.57–1.61 (m, 4H), 1.63–1.71 (m, 4H), 2.06 (s, 3H), 3.42 (t, *J* = 5.7 Hz, 2H), 3.6 (t, *J* = 6.0 Hz, 2H), 3.64–3.75 (4H, m); ^{13}C NMR (125 MHz): δ = 21.84, 30.01, 33.93, 34.80, 39.21, 44.10, 62.30, 99.70, 169.14; GCMS: *m*/*z* (%) = 213 (40) [M^+], 185 (20), 170 (10), 140 (100), 127 (39),113 (25), 98 (32);); anal. calcd. for $C_{11}H_{19}NO_3$: C 61.95, H 8.98, N 6.57; found C 61.79, H 9.26, N 6.61.

1-(2.3-Dimethyl-1,4-dioxa-8-aza-spiro[4.5]decan-8-yl)ethan-1-one (**8c**):

Prepared from a diastereomeric mixture of 2,3-butanediol. Yield: 92%; IR (neat): $\tilde{\nu}$ = 2973, 1637, 1438, 1352, 1244, 1109, 1048 cm^{-1}; ^{1}H NMR (500 MHz): δ = 1.08 (d, *J* = 6.0, 6H), 1.55–1.68 (m, 4H), 2.02 (s, 3H), 3.40–3.49 (m, 2H), 3.56–3.65 (m, 2H), 4.14–4.22 (m, 2H); ^{13}C NMR (500 MHz): δ = 15.93, 21.80, 35.06, 39.79, 44.69, 74.61, 105.87, 169.11; GCMS: m/z (%) = 213 (40) [M^{+}], 158 (25), 127 (100), 112 (15), 98 (25); anal. calcd. for $C_{11}H_{19}NO_3$: C 61.59, H 8.98, N 6.57; found C 61.32, H 8.80, N 6.28.

1-(8,11-Dimethyl-7,12-dioxa-3-aza-spiro[5.6]dodecan-3-yl)ethan-1-one (**9c**):

Prepared from a distereomeric mixture of 2,5-hexanediol. Yield: 67%; IR (neat): $\tilde{\nu}$ = 2968, 2930, 1630, 1446, 1350, 1245, 1120, 1079, 1054 cm^{-1};^{1}H NMR (500 MHz): δ = 1.14 (d, *J* = 6.3 Hz, 6H), 1.51–1.79 (m, 8H), 2.07 (s, 3H), 3.44 (t, *J* = 6.0 Hz, 2H), 3.52–3.69 (m, 2H), 3.93–4.02 (m, 2H); ^{13}C NMR (125 MHz): δ = 21.86, 23.25, 34.07, 36.60, 39.54, 44.41, 70.00, 98.78, 169.16; GCMS: *m*/*z* (%) = 241 (30) [M^{+}], 198 (15), 155 (15), 140 (100), 125 (30), 116 (35), 113 (60), 98 (65); anal. calcd. for $C_{13}H_{23}NO_3$: C 64.70, H 9.61, N 5.80; found: C 64.45, H 9.69, N 5.50.

1-[2R,3R]-2,3-Diphenyl-1,4-dioxa-8-azaspiro[4.5]decan-8-yl]ethan-1-one (**10c**):

Prepared from (*R*,*R*)-1,2-diphenyl-1,2-ethanediol. Yield: 40%; $[\alpha]_D^{20}$= –57.2 (*c* = 1, ethanol); IR (KBr): $\tilde{\nu}$ = 3029, 2946, 2877, 1638, 1432, 1350, 1268, 1249, 1126, 1075, 765, 700 cm^{-1}; ^{1}H NMR (500 MHz): δ = 1.84–2.02 (m, 4H), 2.08 (s, 3H)), 3.59 (t, *J* = 5.7 Hz, 2H), 3.76 (t, *J* = 5.7 Hz, 2H), 4.71 (s, 2H), 7.11–7.16 (m, 4H), 7.23–7.29 (m, 6H); ^{13}C NMR (125 MHz): δ = 21.88, 36.56, 37.65, 39.86, 44.69, 85.79, 108.00, 127.09,

128.93, 136.65, 169.33; GC-MS: *m*/*z* (%) = 337 (3) [M^+], 231 (100); anal. calcd. for $C_{21}H_{23}NO_3$: C 74.75, H 6.87, N 4.15; found C, 74.63, H 7.15, 4.31

1-(Spiro[1,3-benzodioxole-2,4'-piperidine]-1-yl)ethan-1one (**11c**):

Prepared from catechol. Yield: 65%; mp. 153.1–153.9°C; IR (KBr): $\tilde{\nu}$ = 2973, 1645, 1486, 1353, 1237, 1046, 748 cm^{-1};^{1}H NMR (500 MHz): δ = 1.91 (br. s, 4H), 2.09 (s, 3H), 3.59 (s, 2H), 3.75 (s,2H), 6.69–6.77 (m,4H); ^{13}C NMR (125 MHz): δ = 21.86, 35.05, 39.16, 105.24, 115.83, 121.88, 147.17, 169.30; GCMS: *m*/*z* (%) = 233 (100) [M^+], 190 (12), 147 (44), 135 (40); anal. calcd. for: $C_{13}H_{15}NO_3$: C 66.94, H 6.48, N 6.00; found C 67.31, H 6.78, N 6.16.

1-(5,6-Diphenylspiro[1,3-benzodioxole-2,4'-piperidine]-1-yl)ethan-1one (**12c**):

Prepared from 1,2-dihydroxy-4,5-diphenylbenzene. Yield: 53%; mp 242.6–243.7 °C; IR (KBr): $\tilde{\nu}$ = 3422, 3057, 2866, 1648, 1483, 1438, 1349, 1237, 1024, 878, 772, 704 cm^{-1}; ^{1}H NMR (500 MHz): δ = 2.08 (s, 4H), 2.18 (s,3H), 3.70 (s, 2H), 3.86 (s, 2H), 6.86 (s, 2H), 7.05–7.10 (m, 4H), 7.15–7.22 (m, 6H); ^{13}C NMR (125 MHz): δ = 21.88, 39.21, 43.99, 111.16, 116.77, 126.67, 128.26, 130.40, 134.71, 141.82, 146.74, 169.38; EIMS: *m*/*z* (%) = 385 (83) [M^+], 314 (13), 307 (20), 299 (26), 287 (11), 262 (19), 207 (24), 125 (16), 99 (21), 85 (12); anal. calcd. for: $C_{25}H_{23}NO_3$: C 77.90, H 6.01, N 3.63; found: C 77.84, H 6.12, N 3.74.

1-(1,5-Dithia-9-azaspiro-[5.5]uncan-9-yl)ethan-1-one (**13c**):

Boron trifluoride etherate (1.2 mL) was added dropwise to a well-stirred mixture of *N*-acetyl-4-piperidone (1.4 g, 10 mmol) and 1,3-propanedithiol (1.08 g, 10 mmol) at room

temperature. After stirring for another 2 h, water was added carefully, and the mixture was extracted with ethyl acetate (3 × 25 mL). The combined organic layers were dried with sodium sulfate. The crude product obtained after evaporation of the solvent was purified by column chromatography (ethyl acetate) to give the solid, colorless product **13c**. Yield: 1.27 g (69%); R_f = 0.13; mp 91.7–92.2°C; IR (KBr): $\tilde{\nu}$ = 2925, 2887, 1635, 1429, 1275, 1239, 992 cm^{-1}; ^1H NMR (500 MHz): δ = 1.92–2.02 (m, 6H), 2.02–2.04 (m,3H), 2.72 (m, 4H), 3.47–3.68 (4H, m, 4H); ^{13}C NMR (125 MHz): δ = 21.85, 32.02, 34.75, 38.54, 43.41, 59.81, 96.45, 169.27; GCMS: *m*/*z* (%) = 231 (93) [M^+], 198 (28), 188 (17), 156 (42), 146 (14), 124 (100), 114 (52), 106 (14); anal. calcd. for $C_{10}H_{17}NOS_2$: C 51.91, H 7.41, N 6.05; found C 52.02, H 7.24, N 6.03.

General procedure for the N-arylation of *N*-Boc-4-arylaminopiperidines 17 and 18:

A 25 mL two-necked flask, equipped with a magnetic stirrer and a connection to a combined argon/vacuum line was charged with piperidine **19** or **20** (3.6 mmol), the corresponding aryl bromide (3.6 mmol), sodium *tert*-butoxide (0.43 g, 4.5 mmol) and palladium acetate (10.1 mg, 0.045 mmol). The flask was closed with a septum and the air in the flask was replaced by nitrogen. Dry toluene (5 mL) was added, and the mixture was stirred at room temperature for 30 min. Then, a solution of tri-*t*-butylphosphane (0.12 mL, 0.5 mmol) in toluene (1 mL) was added, and the mixture was heated under reflux for 16 h under argon. After cooling to room temperature, water (10 mL) was added, and the organic layer was separated, dried with sodium sulfate and evaporated. The crude product was purified by column chromatography (ethyl acetate/cyclohexane, 1:2) to give the yellowish solid products.

According to this procedure, the following were obtained:

***tert*-Butyl 4-[diphenylamino]piperidine-1-carboxylate** (**2c**):

Prepared from *tert*-butyl 4-(phenylamino)piperidine-1-carboxylate and bromobenzene. Yield: 95%. The spectroscopic data are in accordance with those described in the literature [21].

***tert*-Butyl 4-[bis(4-methylphenyl)amino]piperidine-1-carboxylate** (**3c**):

Prepared from **17** and 4-methylbromobenzene. Yield: 76%; IR (KBr): $\tilde{\nu}$ = 2966, 2935, 2862, 1694, 1509, 1426, 1295, 1134, 1086, 807 cm^{-1}; ^1H NMR (500 MHz): δ = 1.25–1.37 (m, 2H), 1.42 (s, 9H), 1.95 (d, *J* = 11.7 Hz, 2H), 2.31 (s, 6H), 2.74–2.87 (m, 2H), 3.93 (tt, *J* = 11.7 Hz, J = 3.5 Hz, 1H), 4.17 (br. s, 2H), 6.73 (d, *J* = 8.2 Hz, 4H), 7.07 (d, *J* = 8.5, 4H); ^{13}C NMR (125 MHz): δ = 21.05, 28.82, 31.03, 55.60, 79.91, 123.09, 130.26,131.70, 144.16, 155.04; EIMS: *m*/*z* (%): = 380 (45) [M^+], 324 (16), 279 (14), 236 (13), 222 (13), 197 (79), 181 (16), 127 (22), 84 (25), 57 (100); anal. calcd. for: $C_{24}H_{32}N_2O_2$: C 75.75, H 8.48, N 7.36; found C 75.97, H 8.27, N 7.24.

***tert*-Butyl 4-[Bis(4-fluorophenyl)amino]piperidine-1-carboxylate** (**4c**):

Prepared from **18** and 4-fluorobromobenzene. Yield: 41%. IR (KBr): $\tilde{\nu}$ = 3385, 2928, 1686, 1560, 1509, 1421, 1365, 1145, 819, 769 cm^{-1}. ^1H NMR (500 MHz): δ = 1.25–1.34 (m, 2H), 1.42 (s, 9H), 1.92 (d, *J* = 11.4 Hz, 2H), 2.75–2.86 (m, 2H), 3.87 (tt, *J* = 11.7 Hz, *J* = 3.5 Hz, 1H), 4.18 (br. s, 2H), 6.73–6.79 (m, 4H), 6.97 (t, *J* = 8.5 Hz, 4H). ^{13}C NMR (125 MHz): δ = 27.35, 29.58, 54.64, 78.66, 114.96 (d, *J* = 22.9 Hz), 123.90 (d, *J* = 8.3 Hz), 131.70 (d, *J* = 2.8 Hz), 153.58. EIMS: *m*/*z* (%) = 388 (1) [M^+], 205 (4), 84 (12), 57 (100).

General procedure for the N-acyl deprotection of 6c–13c to 6b–13b:

The amides **5c–15c** (4.00 mmol) were dissolved in 60 mL of a mixture consisting of ethanol (150 mL), water (50 mL), and potassium hydroxide (15 g) and then heated under reflux for 20 h. The solvent was removed in a rotary evaporator and the residue was dissolved in ethyl acetate (20 mL), washed with de-ionized water (3 × 40 mL), and dried with sodium sulphate. The crude products obtained after the evaporation of the solvent were used in the following step without further purification. According to this procedure, the following were obtained:

1,5-Dioxa-9-azaspiro[5,5]undecane (**6b**): Yield: 70%.

7,12-Dioxa-3-azaspiro[5,6]dodecane (**7b**): Yield: 96%.

2,3-Dimethyl-1,4-dioxaspiro[4,5]decane (**8b**): Yield: 48%.

8,11-Dimethyl-7,12-dioxa-3-azaspiro[5,6]dodecane (**9b**): Yield: 55%.

(2*R*,3*R*)-2,3-Diphenyl-1,4-dioxaspiro[4,5]decane (**10b**): Yield: 43%; $[\alpha]_D^{20}$ –47.8 (*c* = 1, ethanol).

Spiro[1,3-benzodioxole-2,4'-piperidine] (**11b**): Yield: 98%.

5,6-Diphenylspiro[1,3-benzodioxole-2,4'-piperidine] (**12b**): Yield: 88%.

1,5-Dithia-9-azaspiro[5,5]undecane (**13b**): Yield: 48%.

1,4-Dioxa-8-azaspiro[4,5]decane (**5b**) is commercially available and was purchased.
